# Evaluation of Proxy Tests for SFSN: Evidence for Mixed Small and Large Fiber Dysfunction

**DOI:** 10.1371/journal.pone.0042208

**Published:** 2012-08-03

**Authors:** Hamid Ebadi, Bruce A. Perkins, Hans D. Katzberg, Leif E. Lovblom, Vera Bril

**Affiliations:** 1 Division of Neurology, Department of Medicine, University Health Network, Toronto, Ontario, Canada; 2 Division of Metabolism and Endocrinology, Department of Medicine, University Health Network, Toronto, Ontario, Canada; Charité University Medicine Berlin, Germany

## Abstract

**Background:**

Though intra-epidermal nerve fiber density (IENFD) is considered the gold standard for diagnosis of small fiber sensory neuropathy (SFSN), we aimed to determine if novel threshold values derived from standard tests of small or large fiber function could serve as diagnostic alternatives.

**Methods:**

Seventy-four consecutive patients with painful polyneuropathy and normal nerve conduction studies (NCS) were defined as SFSN cases or controls by distal IENFD <5.4 and ≥5.4 fibers/mm, respectively. Diagnostic performance of small fiber [cooling (CDT) and heat perception (HP) thresholds, axon reflex-mediated neurogenic vasodilatation] and large fiber function tests [vibration perception thresholds (VPT) and sural nerve conduction parameters] were determined by receiver operating-characteristic (ROC) curve analyses.

**Results:**

The 26(35%) SFSN cases had mean IENFD 3.3±1.7 fibers/mm and the 48(65%) controls 9.9±2.9 fibers/mm. Male gender (p = 0.02) and older age (p = 0.02) were associated with SFSN cases compared to controls. VPT were higher and CDT lower in SFSN cases, but the largest magnitude of differences was observed for sural nerve amplitude. It had the greatest area under the ROC curve (0.75) compared to all other tests (p<0.001 for all comparisons) and the optimal threshold value of ≤12 µV defined SFSN cases with 80% sensitivity and 72% specificity.

**Conclusion:**

In patients presenting with polyneuropathy manifestations and normal NCS, though small fiber function tests were intuitively considered the best alternative measures to predict reduced IENFD, their diagnostic performance was poor. Instead, novel threshold values within the normal range for large fiber tests should be considered as an alternative strategy to select subjects for skin biopsy in diagnostic protocols for SFSN.

## Introduction

Small fiber sensory neuropathy (SFSN) is an axonal neuropathy involving mainly thinly myelinated (Aδ) and unmyelinated (C) fibers [1], [2]. Patients with SFSN may have sensory symptoms of neuropathic pain, autonomic symptoms or abnormal thermal sensations, but sensory symptoms comprise the most common clinical presentation. The traditional evaluation of polyneuropathy is centered on clinical assessment and measures of large nerve fibers, i.e.: nerve conduction studies (NCS) and vibration perception thresholds (VPT) [3]. SFSN is suspected in the situation in which patients present with normal NCS and VPT, and diagnosis requires specialized approaches compared to the classical evaluation of polyneuropathy [4]. In the last decade, intra-epidermal nerve fiber density (IENFD) obtained from skin punch biopsy specimens has become widely accepted as one of the most accurate diagnostic methods for SFSN [4], [5] and has become the gold standard for confirmatory diagnosis of SFSN in a clinical guideline. [4] Particularly if age- and gender-delineated thresholds are used, the method is highly sensitive and specific for the diagnosis of SFSN [6], [7]. Although the skin punch biopsy is simple to perform, it remains an invasive procedure and assessment of IENFD is a costly investigation that requires enormous lab resources and expertise that are not available in most clinics. In view of this, it is essential to determine if more broadly available and non-invasive functional tests can serve as proxy measures of IENFD. Candidate tests include small fiber functional measures such as quantitative thermal sensory thresholds (QST) of heat-pain [HP] and cooling-detection [CDT] and reflex-mediated neurogenic dilatation [laser Doppler flare imaging (LDI)] tests. Furthermore, large fiber sensory tests such as sural nerve electrophysiological parameters may have diagnostic performance for SFSN if alternative threshold values to those published for healthy populations are considered.

We aimed to determine in a group of patients presenting with polyneuropathy symptoms, but with normal large fiber function tests, whether small fiber tests of QST and LDI or large fiber sural nerve electrophysiological tests could serve as proxy measures for IENFD for the diagnosis of SFSN.

## Methods

A retrospective chart review of patients referred to the neuromuscular clinic of Toronto General Hospital during the years 2008–2011 for suspected SFSN was done. The Research Ethics Board of the University Health Network approved the study.

All of the patients had a detailed neurological history, completed an 11-point Likert rating scale for mean pain intensity, and underwent physical examination including examination of all primary sensory modalities; namely: pin prick sensation, temperature, light touch, vibration and proprioception. NCS, VPT, quantitative thermal threshold tests of HP and CD, LDI, and IENFD were performed in all subjects.

The assessment of pain was determined using an 11-point Likert visual analog scale of the modified short form McGill pain questionnaire [8].

Patients were included if they had suspected idiopathic SFSN. Those with underlying causes of SFSN were excluded; namely: diabetes, impaired glucose tolerance, vitamin B12 deficiency, alcoholism, family history, neoplasia, uremia, history of toxic drug exposure, etc. One patient has a faint IgG kappa paraprotein band that was thought to be unrelated to the symptoms. All others had normal serum immunoelectrophoresis.

### Large Fiber Function Testing

For NCS, testing involved antidromic examination of unilateral sural nerves. Two sural parameters were studied, the sural nerve action potential amplitude and the sural nerve conduction velocity. The test was performed using the Sierra Wave NCS equipment (Cadwell Laboratories Inc, Kennewick, WA, USA) meeting the standards of the American Association for Neuromuscular and Electrodiagnostic Medicine and the Canadian Society of Clinical Neurophysiology.

Quantitative measurement of VPT was performed with the Horwell Neurothesiometer (Scientific Laboratory Supplies Ltd, Hessle, Yorskshire, UK) on the right great toe and right index finger using the method of limits procedure [9]. For each trial, the vibration stimulus started at zero, and was gradually increased until the patient reported feeling the vibration. In addition, we did a catch trial, when no vibrating stimulus was presented. Three vibrating trials were performed per testing, and the final result was a mean of the three trials. The intensity and the speed of intensity change were deliberately irregular.

### Small Fiber Function Testing

The assessment of axon–reflex mediated neurogenic vasodilatation in response to cutaneous heating was done by the LDI technique using the moorLDI2™ (Moor Instruments Ltd, Axminster, UK) [10]. The surface skin temperature of the dorsum of the foot was standardized to 32°C using a warm blanket. We subsequently used a standard skin-heating probe (Moor Instruments Ltd, Axminster, UK), a 0.64 cm^2^ circular metal disc that was well-affixed to the skin above the first metatarsal area on the dorsum of the foot, that heated the skin to 44°C for 20 minutes. After probe removal the cutaneous flare was measured by LDI. The laser head of the LDI apparatus was positioned at a fixed distance of 30 cm from the dorsum of the foot and scanned an area of 6 cm×6 cm (36 cm^2^). The LDI apparatus used a scanning Doppler infrared laser beam with a wavelength of 785 nm, sufficient to penetrate skin to register the movement of blood cells in dermal capillaries. The 36 cm^2^ area represented a 256×256 pixel resolution with each pixel itself representing a measurement of the velocity of tissue blood flow. The total scanning time was less than five minutes per examination. The flare area (cm^2^) was calculated using Moor LDI software (version 3.11).

CDT was obtained using the TSA-II NeuroSensory Analyzer (Medoc Advanced Medical Systems, Ramat-Yishai, Israel). The CDT was tested using a method of limits [9]. The stimulator was applied to the dorsum of the foot and hand at a temperature of 32°C and the temperature was gradually decreased to the first level detected by the patient as a cooler stimulus than the preceding. Five trials were performed and a catch trial, with null stimulus, was inserted randomly during testing. An average of the five levels was taken for each of the studies on the foot and hand.

### Classification of SFSN Cases and Controls

The patients were classified as SFSN cases or controls based on IENFD with SFSN having IENFD <5.4 and normal subjects having SFSN ≥5.4/mm (Therapath LLC, NCCLS, NY, NY). A single threshold value, not considering age or gender adjustments, was used according to the commercial test interpretation. This approach is unlikely to influence the prediction of IENFD results by either large or small fiber function tests. 3 mm punch biopsies were performed just proximal to the lateral malleolus, fixed in 2% PLP and sent to Therapath for analyses. H&E and PGP 9.5 staining was performed and the IENFD obtained by using established counting rules. [11], [12]. The analyses were done by counting the number of epidermal fibers that cross the basement membrane, counting 5 separate tissue sections. The total number of fibers was divided by the length of the epidermis in the five sections, giving the IENFD (fibers per mm length of epidermis). The IENFD was compared to the values in specimens from normal control subjects (Therapath).

Laboratory testing for metabolic, immune, infectious, and endocrine (including 2 hour OGTT) causes of neuropathy were performed in all patients. All relevant demographic and test data were abstracted from clinical records.

### Statistical Analysis

All statistical analyses were performed in SAS version 9.3 (SAS Institute, Carey, North Carolina). Student’s t-tests were used to compare continuous data between SFSN cases and controls while χ2 tests were performed for categorical data. P values <0.05 were considered significant. Receiver Operating Characteristic (ROC) curve analysis was used to determine the operating characteristics of small and large fiber tests on the identification of SFSN cases and controls. Area under the ROC curves for each of the tests were compared according to the method of Pencina et al. Specifically, two-tailed p-values were calculated using a z-score obtained from testing the hypothesis that the areas under two different ROC curves are the same. Optimal threshold values were obtained by calculating the shortest distance between each variable’s ROC curve and the upper left hand corner of the ROC graph in which sensitivity and specificity are 100%, according to the distance formula for two points in the plane, 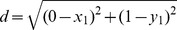. According to the threshold values of the tests, we aimed to report the sensitivity and specificity, as well as the predictive values of the optimally-performing large or small fiber tests. The predictive value positive was defined by the proportion of SFSN cases in subjects with an abnormal (positive) test result and thus represented the probability of having SFSN in a subject with an abnormal small or large fiber test result. The predictive value negative was defined by the proportion of controls without SFSN in subjects with a negative test result and thus represented the probability of not having SFSN in a subject with a negative small or large fiber test result.

## Results

Seventy-four consecutive patients who were evaluated for polyneuropathy symptoms, had normal NCS, and who underwent IENFD were recruited for the chart review. The clinical characteristics for the 74 subjects are shown in Table 1 according to SFSN cases and controls with normal IENFD. Duration of symptoms or degree of pain recorded by VAS did not differ between cases and controls, but cases were older (p = 0.02) and more frequently male (p = 0.02). Primary sensory modalities, including the small fiber modalities of temperature and pinprick, and deep tendon reflexes did not differ between cases and controls. Among the large fiber function tests, VPT obtained on the foot was significantly higher, while sural nerve amplitude and conduction velocity were significantly lower among cases as compared to controls (Table 1). Of the small fiber tests, CDT of the foot was significantly lower in cases.

**Table 1:** Characteristics of the 72 Patients with Clinical Polyneuropathy and Normal Large Fiber Tests According to Presence and Nature of Small Fiber Neuropathy.

Clinical Characteristic	Normal	Small Fiber Sensory Neuropathy	P-values*
	(IENFD≥5.4)	(IENFD <5.4)	
	(n = 48)	(n = 26)	
Age (yr)	49.2±13.1	57.0±12.9	0.015
Female Gender (%)	30 (63%)	9 (35%)	0.02
Duration of symptoms (y)	5.6±5.0	4.3±2.6	0.22
			
Visual Analogue Scale for Pain (cm)	7.0±2.2	7.3±2.2	0.59
Sensory Examination Abnormal	35 (73%)	22 (85%)	0.24
Abnormal Pin Prick Exam	25 (52%)	13 (50%)	0.86
Abnormal Temperature Exam	34 (71%)	20 (77%)	0.57
Abnormal Light Touch Exam	27 (56%)	17 (65%)	0.44
Abnormal Vibration Exam	19 (40%)	13 (50%)	0.39
Abnormal Position Exam	4 (8%)	3 (12%)	0.66
Abnormal Deep Tendon Reflexes	9 (19%)	6 (23%)	0.66
IENFD	9.9±2.9	3.3±1.7	<0.0001
Large Fiber Tests			
Vibration Perception Threshold			
Toe	9.7±3.4	12.5±5.8	0.015
Finger	3.9±1.1	4.3±1.0	0.13
Sural Nerve Amplitude (µV)	16.0±7.4	10.5±5.9	0.002
Sural Nerve Conduction Velocity (m/s)	49.9±4.9	47.0±5.6	0.02
Small Fiber Tests			
CDT Foot (°C)	26.7±6.4	21.6±9.1	0.007
CDT Hand (°C)	28.4±3.6	27.2±6.3	0.30
HP Foot (°C)	46.9±3.4	48.3±2.9	0.09
HP Hand (°C)	45.2±5.1	46.4±4.2	0.31
LDI flare (cm)	2.3±1.2	2.1±1.1	0.59

* P values for dichotomous variables were calculated with the χ2 test and t-test was used for continuous variables.

For VPT, the normal values are highly age-dependent, but values ≤5 are normal in the finger and ≤15 Volts are normal in the toe. For VPT, data are available in 42 patients with normal IENFD and on 24 patients with SFSN.

Cut-offs for quantitative sensory thresholds are age-dependent although, generally a normal CDT would be ≥25°C. The normal values for heat pain are ≤50°C.

**Figure 1:**
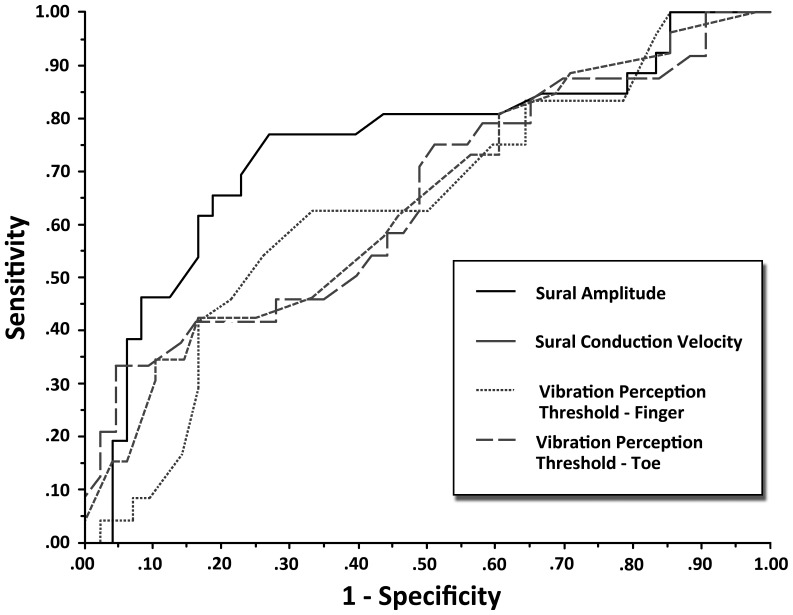
Shows the ROC curves for large fiber function tests of sural nerve amplitude and conduction velocity and vibration perception thresholds at index finger and first toe. The curve for sural nerve amplitude lies closest to the upper y-axis and has the largest AUC at 0.75.

**Figure 2:**
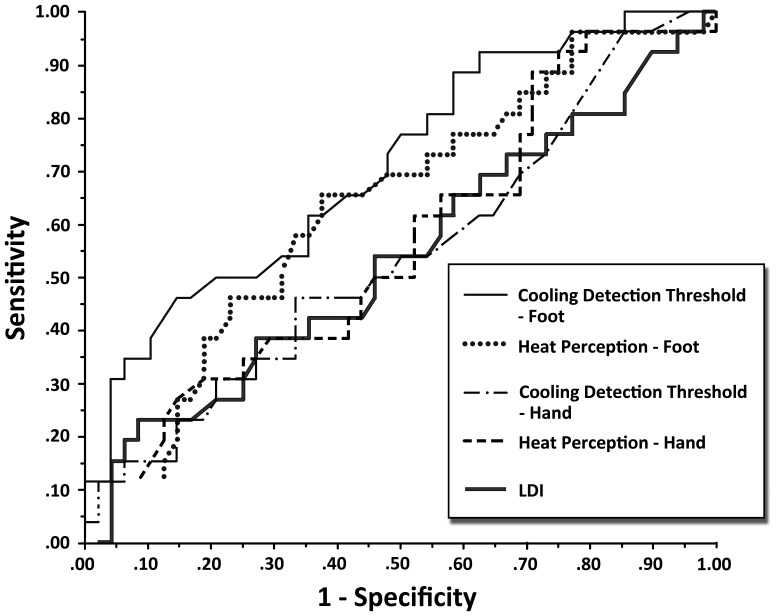
Shows the ROC curves for small fiber function tests of cooling detection thresholds and heat perception thresholds in upper and lower extremities and the laser Doppler flow studies in the foot.

**Figure 3:**
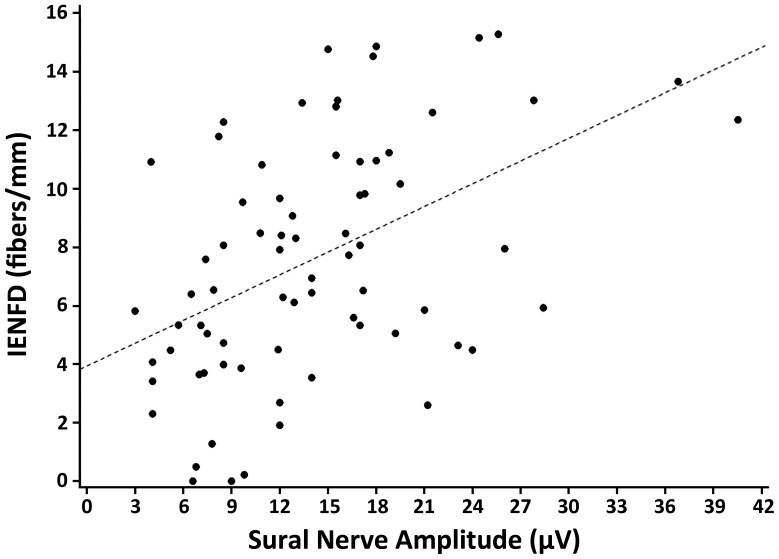
Shows the linear regression model for IENFD as a function of the sural sensory nerve action potential amplitude. (R^2^ = 0.22, p<0.001).

To determine if the differences observed between cases and controls for the large and small fiber tests could have diagnostic accuracy for identification of SFSN, we present the ROC curves for large fiber tests in Figure 1 and the small fiber tests in Figure 2. Table 2 summarizes the AUC and operating characteristics of the optimal threshold values for the tests. As seen in Figure 1, the large fiber test with the largest AUC was the sural nerve amplitude. For this parameter, area under the curve was 0.75, and the optimal threshold value that maximized sensitivity (0.77) and specificity (0.73) for detection of SFSN was ≤12.0 µV (Table 2, Figure 3). Compared to the other large fiber tests, the AUC was significantly greater than that of sural nerve conduction velocity and VPT at the hand, but not significantly greater than VPT at the foot. However, its optimal threshold level had substantially greater sensitivity and specificity than that of VPT at the foot.

**Table 2:** Comparison of the Area Under the Receiver Operating Characteristic Curve and Optimal Thresholds for Sural Nerve Amplitude and the Other Nerve Fiber Function Tests.

				Operating chart of the optimal threshold values **	
Test	Area under theROC curve	P Value*	Optimal threshold for SFSN case definition	Sensitivity	Specificity
Sural nerve amplitude†	0.75	–	≤12.0 µV	0.77	0.73
Large Fiber Tests					
Sural nerve CV	0.64	0.01	≤48.3 m/s	0.62	0.54
VPT Foot	0.65	0.15	≥9.2	0.71	0.51
VPT Hand	0.64	0.004	≥4.2	0.63	0.67
Small Fiber Tests					
CDT Foot	0.71	0.19	≤26.2°C	0.62	0.65
CDT Hand	0.54	<0.0001	≤28.9°C	0.46	0.67
HP Foot	0.63	0.004	≥48.7°C	0.65	0.63
HP Hand	0.56	<0.0001	≥47.0°C	0.62	0.48
LDI	0.54	<0.0001	≤1.96 cm^2^	0.54	0.54

* Two-tailed p values were calculated using a z-score obtained from testing the hypothesis that the areas under two different ROC curves are the same, according to the method of Pencina et al.

† P value not applicable for sural nerve amplitude as it used as the reference to which the other tests are compared.

** Optimal values were obtained by calculating the shortest distance between each variable’s ROC curve and the upper left hand corner of the ROC graph, according to the distance formula for two points in the plane, 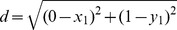.

The ROC curves for the small fiber function tests are shown in Figure 2. Compared to that of sural nerve amplitude, all of these had significantly lower area under the curve except for CDT at the foot (Table 2). However, the optimal threshold value for sural nerve amplitude was associated with substantially greater sensitivity and specificity than that of CDT at the foot (Table 2).

We calculated predictive values and overall diagnostic accuracy for SFNP as the parameter with a threshold associated with the highest sensitivity and specificity. The negative predictive value of the sural nerve amplitude >12 µV was 85% and the positive predictive value of sural nerve amplitude of ≤12 µV was 61%. Overall diagnostic accuracy for this threshold was 74%.

## Discussion

In this cohort of patients with symptoms suggestive of polyneuropathy and normal large fiber function tests, though we observed that variables such as older age, male gender and lower CDT were associated with SFSN cases, the variables most prominently associated with cases were the large fiber function tests of VPT and sural nerve electrophysiology. Though it had some limitations in its ability to detect SFSN cases, sural nerve amplitude had the best diagnostic performance even compared to small fiber function tests. Receiver operating characteristic curve analysis provided the rationale for a working threshold value for sural nerve amplitude of ≤12 µV, well within the normal range of sural nerve amplitudes, as it had optimal sensitivity and specificity. According to its negative predictive value, patients with polyneuropathy symptoms, normal NCS, but a sural nerve amplitude value above this threshold were 85% likely to have normal IENFD and be classified as controls without SFSN. However, those with sural nerve amplitude ≤12 µV, but still within the normal range, were only 61% likely to be classified as SFSN cases. The clinical implication of this finding is that a non-invasive large fiber measure could potentially be incorporated into a protocol that could stratify patients for consideration of skin biopsy for IENFD.

The current management of patients presenting with painful polyneuropathy symptoms and normal NCS is to refer them for small nerve fiber function tests and IENFD assessment although the strength of the recommendations for IENFD is controversial [4], [13]. The current research implies that a potential protocol that could stratify patients could help to decrease the frequency of invasive and expensive biopsy. Specifically those with levels above the diagnostic threshold could be considered to have low pre-test probability of SFSN and therefore not be subjected to biopsy. Such a protocol could prevent biopsy in more than half of individuals who would ordinarily be subjected to the diagnostic procedure. Determination of the precise threshold values and testing of such protocols requires further study.

Different methods with variable performance characteristics have been developed to investigate small nerve fiber function. LDI [14], quantitative thermal sensory threshold testing for perception of heat and cold stimuli [15], autonomic testing of the quantitative sudomotor axon reflex [16], cardiovascular autonomic function testing, sympathetic skin response, laser evoked potentials [17] and contact heat evoked somatosensory potentials [18], [19] are some of these methods. To date, none of them have become standard practice due to limitations in performance and they are confined mainly to research protocols. Few studies have compared IENFD with small fiber function tests [4], [20], [21]. This study suggest the view that existing small fiber nerve function tests are very limited in diagnostic performance for identifying SFNP confirmed by skin biopsy. Though cooling-detection thresholds had an acceptable area under the ROC, they lacked a single discriminatory threshold to classify cases and controls. Similarly, no diagnostic advantage was observed with heat-pain tests or axon–reflex mediated neurogenic vasodilatation in response to cutaneous heating.

NCS measure large fiber function accurately. The sural nerve amplitude is a measure of the number of large fibers and the conduction velocity measures the speed that nerve impulses travel along the fastest, and largest fibers in the nerve. With polyneuropathy affecting large fibers, the amplitudes of nerve action potentials decline as nerve fibers are lost [22]. The sural nerve conduction velocity decreases with loss of the largest fibers. Although a good measure of large nerve fiber function, the prevailing concept is that NCS are insensitive to small fiber function and loss. The findings in the current study reveal a relationship between mild degrees of large fiber dysfunction, and small fiber loss that can help in the diagnosis of SFSN. This indicates that small fibers are not affected purely in isolation, but that they are accompanied by evidence of subtle alterations in large fiber function that fall within published normal ranges. For example, though the sural nerve action potential normal amplitude is >6 µV, here we observe levels of ≤12 µV are associated with SFSN.

Though the current study highlights an important potential role of large fiber tests in the determination of diagnostic protocols for SFSN, it does have limitations. First, it was a retrospectively performed chart-review that is susceptible to selection bias as subjects were clinically selected for IENFD determination. Second, case definition of SFSN was based on a single rather than age-sex-height-adjusted threshold IENFD value. Though the sensitivity of IENFD for SFSN may be affected by this lack of adjustment, it is unlikely that the predictive results of small or large fiber function test for IENFD would be affected by this omission. Finally, we did not evaluate all known tests for small fiber nerve function, such as cardiac autonomic system testing and others listed above, but only an older standard test (HP and CDT) and a novel method, LDI flare.

In patients presenting with polyneuropathy and normal NCS, small fiber function tests had poor diagnostic performance for SFSN, but the large fiber parameter of sural nerve amplitude demonstrated acceptable performance at a threshold in the lower distribution of normal values. Novel threshold values for large fiber tests should be considered as an alternative strategy to select subjects for skin biopsy in diagnostic protocols for SFSN.
